# In Vitro/In Vivo Preparation and Evaluation of cRGDyK Peptide-Modified Polydopamine-Bridged Paclitaxel-Loaded Nanoparticles

**DOI:** 10.3390/pharmaceutics15112644

**Published:** 2023-11-20

**Authors:** Dan Yun, Dengyuan Liu, Jinlin Liu, Yanyi Feng, Hongyu Chen, Simiao Chen, Qingchun Xie

**Affiliations:** 1 Center for New Drug Research and Development, Guangdong Pharmaceutical University, Guangzhou 510006, China; 2 Guangdong Provincial Key Laboratory of Advanced Drug Delivery Systems, Guangdong Pharmaceutical University, Guangzhou 510006, China; 3 Guangdong Provincial Engineering Center of Topical Precision Drug Delivery System, Guangdong Pharmaceutical University, Guangzhou 510006, China

**Keywords:** nanoparticles, RGD, PLGA, surface modification, antitumor effect

## Abstract

Cancer remains a disease with one of the highest mortality rates worldwide. The poor water solubility and tissue selectivity of commonly used chemotherapeutic agents contribute to their poor efficacy and serious adverse effects. This study proposes an alternative to the traditional physicochemically combined modifications used to develop targeted drug delivery systems, involving a simpler surface modification strategy. cRGDyK peptide (RGD)-modified PLGA nanoparticles (NPs) loaded with paclitaxel were constructed by coating the NP surfaces with polydopamine (PD). The average particle size of the produced NPs was 137.6 ± 2.9 nm, with an encapsulation rate of over 80%. In vitro release tests showed that the NPs had pH-responsive drug release properties. Cellular uptake experiments showed that the uptake of modified NPs by tumor cells was significantly better than that of unmodified NPs. A tumor cytotoxicity assay demonstrated that the modified NPs had a lower IC_50_ and greater cytotoxicity than those of unmodified NPs and commercially available paclitaxel formulations. An in vitro cytotoxicity study indicated good biosafety. A tumor model in female BALB/c rats was established using murine-derived breast cancer 4T1 cells. RGD-modified NPs had the highest tumor-weight suppression rate, which was higher than that of the commercially available formulation. PTX-PD-RGD-NPs can overcome the limitations of antitumor drugs, reduce drug toxicity, and increase efficacy, showing promising potential in cancer therapy.

## 1. Introduction

Malignant tumors have become a significant cause of human mortality, meaning that antitumor research and drug development address serious unmet medical needs. Chemotherapy is one of the most common clinical cancer treatments. However, most currently available conventional chemotherapeutic drugs are poorly water-soluble [1] and have poor tissue selectivity [2]. Increasing drug solubility and avoiding systemic toxicity are major challenges for their clinical application, and these limitations are often overcome by using drug delivery systems including nanoparticles. Their biosafety, passive targeting [3], pH-responsiveness [4,5], and sustained and controlled release [6,7,8,9] have made nano-drug delivery systems (NDDS) a hot research topic [2,10,11].

The surface modification of nano-preparations has been used to overcome their limitations [12,13], aiming for medications to actively target cancer cells while reducing the harmful effects of the drugs on healthy cells [14,15]. Sodium hyaluronate, transferrin, folic acid, and peptides are commonly used as targeting ligands. The cRGDyK peptide (RGD) is a peptide that targets the cell-surface integrin receptor. In contrast to normal tissues, integrin αvβ3 receptors [16,17] are expressed on most cancer cells and offer a new target for cancer therapy. RGD-peptide–nanocarrier coupling was utilized to implement active targeted transport [18,19], taking advantage of the differences in receptor expression between normal and cancer tissues. Na et al. [20] reported that RGD- and lactoferrin-bimodified liposomes loaded with docetaxel enhanced the brain-targeting effects and treatment of gliomas. The overexpression of the integrin αvβ3 receptor and lactoferrin receptor in brain microvascular endothelial and glioma cells was exploited to enable modified liposomes to target tumor sites.

The three main active-targeting-ligand binding types are physical binding, chemical bonding, and binding via intermediates. Physical binding usually refers to adding a ligand modifier to a solution via charge adsorption; however, this results in poor binding and little coupling. Although chemical bonding is a frequently used binding technique, it typically involves complicated chemical reactions, consumes large quantities of reagents, and is difficult to follow up with subsequent alterations. Low yields and numerous by-products are characteristic of the process. Using intermediates as a bridge to link nanoparticles (NPs) with active ligands, Shi et al. chemically bound RGD to distearoyl phosphatidylethanolamine–polyethyleneglycol (DSPE-PEG) and subsequently embedded it in polylactic acid-hydroxyacetic acid (PLGA) NPs as an excipient [21,22]. However, these methods are similar to traditional physicochemical surface modification methods. Therefore, it is necessary to develop a simpler and more versatile surface modification strategy to meet the needs of targeted nano-drug delivery systems.

In 2007, Lee et al. [23] found that mussels are strongly adhesive even on wet surfaces and that the main reason for their adhesion is the catechol structure and amino groups of dopamine (DA) in mucin [24]. Polymerized dopamine (polydopamine) also possesses these reactive groups [25,26,27]. This inspired the development of bionanochemistry and surface modification techniques based on polydopamine and other mussel foot proteins, making mussel bionanochemistry a research focus area [28,29]. Active-targeting modifiers such as protein peptides [19,30], folic acid [31], and hyaluronic acid [32] can be attached to surfaces that require modification using the secondary modification characteristics of polydopamine [33,34,35], considerably enhancing the efficacy and purposefulness of drug delivery [36,37].

Unlike traditional NP-targeted ligand physicochemical binding modification strategies, a straightforward and user-friendly polydopamine surface modification method was employed in this study. Paclitaxel (PTX) [38,39] was selected as a model drug [40,41]. Paclitaxel-loaded PLGA NPs were prepared, and their surfaces were modified with a polydopamine coating. The secondary reactivity of polydopamine was exploited to place the targeting ligand, RGD cyclic peptide, on the surfaces of the NPs. Paclitaxel-loaded PLGA NPs modified with polydopamine as the bridging RGD peptide were successfully constructed. Preliminary studies were conducted on the nanomedicine-related properties, biosafety, drug release mechanisms, and in vitro and ex vivo antitumor pharmacodynamic effects.

## 2. Materials and Methods

### 2.1. Materials

Paclitaxel was purchased from Dalian Meilun Biotechnology Co., Ltd. (Liaoning, China). The paclitaxel standard was purchased from the China Biological Products Testing Institute (Beijing, China). Paclitaxel injection was purchased from Chongqing Meilai Pharmaceutical Co., Ltd. (Chongqing, China). PVA 205 was purchased from Kelaoli International Trade Shanghai Co., Ltd. (Shanghai, China). Dopamine was purchased from Sigma-Aldrich Trading Co., Ltd. (Shanghai, China). The cRGDyK cyclopeptide was purchased from Nanjing Peptide Biology Co., Ltd. (Nanjing, China). Polylactic-co-glycolic acid (MW = 10 k, LA–GA copolymerization ratio 75/25, carboxyl capping) was purchased from Guangzhou Zuoke Biotechnology Co., Ltd. (Guangzhou, China). Hematoxylin staining and DAB color development kits were purchased from Wuhan Servicebio Biotechnology Co., Ltd. (batch numbers G1209 and G211, respectively; Wuhan, China). Cell Counting Kit-8 was supplied by Dojindo Molecular Technologies, Inc. (Kumamoto, Japan). All other chemicals, ingredients, and reagents were of analytical grade, the solvents used were HPLC-grade, and double-distilled water was used.

### 2.2. Development of PTX PD-RGD NPs

#### 2.2.1. Development of PTX NPs

The emulsification–solvent-evaporation method was used to prepare paclitaxel-loaded PLGA NPs (PTX NPs). A 2% PVA aqueous solution was prepared as the aqueous phase; 10 mg of paclitaxel and 80 mg of PLGA were added to 2 mL of dichloromethane and 0.2 mL of ethanol and vortexed to complete dissolution to form an organic phase. During probe crushing, the organic phase was injected into the aqueous phase and ultrasonicated in an ice bath for 10 min (power: 150 kHz; variable-amplitude rod: 3 mm; JY88IIN; Ningbo Scientz Biotechnology Co., Ltd., Ningbo, China). The obtained emulsions were spun under reduced pressure in a rotary evaporator at 40 °C for 15 min to evaporate the organic solvent. The NPs were harvested via centrifugation at 8150× *g* for 20 min at 4 °C to remove free paclitaxel.

#### 2.2.2. NP Surface Modification

PTX NPs were modified using a one-pot synthesis method [42,43]. The core NPs were prime-coated with polydopamine (PD) by incubating 20 mg of PTX NPs in 20 mL of dopamine hydrochloride solution in tris buffer (10 mM, pH 8.5) for 3 h at room temperature, with rotation. The dopamine concentration was fixed at 0.5 mg/mL. The thickness of the polydopamine nano-coat was about 2 nm at a dopamine concentration of 0.5 mg/mL [44,45]. The NPs were harvested via centrifugation at 8150× *g* for 20 min at 4 °C to remove free dopamine, yielding PDA-modified PLGA NPs loaded with paclitaxel (PTX-PD-NPs).

**Figure 1:**
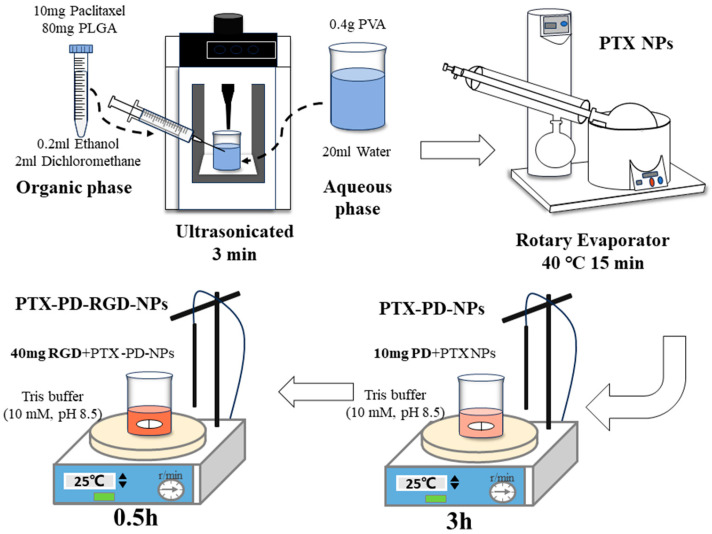
Preparation process of PTX PD-RGD NPs.

The PTX-PD-NPs were resuspended for surface functionalization in Tris buffer (10 mM, pH 8.5) containing cRGDyK ligands. The final concentrations of PTX-PD-NPs and ligands were 1 and 2 mg/mL, respectively. After 30 min of incubation at room temperature (25 °C) with rotation, the particles were collected via centrifugation and washed once with deionized water, yielding cRGDyK-modified PDA-bridged PLGA NPs loaded with paclitaxel (PTX PD-RGD NPs). The preparation process of PTX PD-RGD NPs is shown in Figure 1.

### 2.3. Characterization of PTX PD-RGD NP Formulations

#### 2.3.1. Size and Zeta Analysis

The hydrodynamic particle sizes and surface charges of the three types of NPs (PTX NPs, PTX PD NPs, and PTX PD-RGD NPs) were determined using a laser particle sizer (Delsa Nano C; Beckman Coulter Scientific Instruments Inc., Brea, CA, USA). The dispersion medium was deionized water. The particle size and distribution were determined using dynamic light scattering (DLS), and the surface charge was measured using electrophoretic light scattering (ELS).

#### 2.3.2. Encapsulation Efficiency of NPs

The encapsulation rates of the three NPs were determined using the ultrafiltration–centrifugation method (retention molecular weight of the ultrafiltration tube: 30 kDa; centrifugation temperature: 4 °C). The purified NPs were collected and broken down using acetonitrile. The recovered filtrate was injected into a UPLC system (ACQUITY UPLC H-Class, Waters Technology Inc., Milford, MA, USA) for content determination. The encapsulation efficiency (EE) was calculated as follows:

(1) $$EE(\%)=\frac{C_{Encapsulated\;drugs}}{C_{Total\;dosage}}\times 100\%$$

The chromatographic conditions were as follows: column: ACQUITY UPLC HSS T3 1.8 μm (2.1 × 100 mm); flow rate: 0.5 mL/min; column temperature: 30 °C; injection volume: 2 μL; mobile phase: acetonitrile: water (52:48, *v*/*v*); detection wavelength: 227 nm; retention time: 2.8 min.

#### 2.3.3. Morphological Evaluation

The morphologies of the three NPs were observed using transmission electron microscopy (TEM) (JEM-1400, JEOL, Tokyo, Japan). The NP solution was adsorbed onto a copper grid, stained with 1% phosphotungstic acid solution for 10 min, and observed under a transmission electron microscope at 11 kx and 36 kx magnification.

### 2.4. Solid-State Characterizations

#### 2.4.1. Differential Scanning Calorimetry (DSC) Characterization

Approximately 5 mg of a sample of paclitaxel powder, a physical mixture of paclitaxel and excipients (PM), and paclitaxel NP powder were sealed in aluminum pans and analyzed using a differential scanning calorimeter (DSC4000, PerkinElmer, Waltham, MA, USA) at an increasing temperature rate of 10 °C/min under dry nitrogen gas.

#### 2.4.2. Fourier Transform Infrared (FTIR) Characterization

The structures of blank PLGA NPs, blank PLGA-PD NPs, blank PLGA PD-RGD NPs, PTX, PM, and PTX PD-RGD NPs were analyzed using FTIR (IS5, Thermo Fisher Scientific, Waltham, MA, USA). Dried KBr powder (100 mg) and 3 mg of test sample powder were mixed, ground, pressed, and measured at the scanning wavelength range of 400–4000 cm^−1^. Dried KBr was used as a blank.

#### 2.4.3. X-ray Photoelectron Spectroscopy (XPS) Characterization

XPS was used to analyze the surface elements of the three blank NPs: PLGA NPs, PLGA PD NPs, and PLGA PD-RGD NPs. The three types of NPs were lyophilized after centrifugal ultrafiltration and washing with water, and the lyophilized NP powder was collected for XPS (K-Alpha, Thermo Fisher Scientific, Waltham, MA, USA) analysis.

### 2.5. In Vitro Drug Release Study of PTX from NPs

Reverse dialysis was used to determine the in vitro release behavior of the three different coat-modified nanomedicines. Each NP (PTX NPs, PTX PD NPs, or PTX PD RGD NPs; 3 mL each) was placed in phosphate buffer containing 30 mL of 1% SDS [5,46] (pH = 7.4, 6.8, and 5.2, respectively) and kept at a constant temperature (37 ℃) and oscillation (100 rpm). At predetermined time points (0.5, 1, 2, 4, 6, 8, 12, 24, 48, 72, 96, 120, and 144 h), 5 mL samples were withdrawn, replaced with the same volume of fresh medium, and analyzed using UPLC. Each experiment was performed in triplicate.

(2) $$Q_{n}=\frac{VC_{n}+\sum_{n-1}5C_{n-1}}{m}\times 100\%$$

where Q_n_ is the cumulative drug release rate, %; V is the total volume of the release medium, 30 mL; C_n_ is the drug concentration in the release medium at the nth sampling, μg/mL; m is the total amount of the drug encapsulated by NPs, μg; and n is the sampling time.

### 2.6. Cell Lines

The Guangdong Provincial Key Laboratory of Advanced Drug Delivery provided the 4T1, A549, and HeLa cells. Cells were grown in glass culture flasks for the experiments and used during the exponential growth phase. Fetal bovine serum, antibodies, RPMI1640 medium, and Dulbecco’s modified Eagle’s medium (DMEM) were supplied by GIBCO (C11995500BT, Billings, MT, USA).

### 2.7. Cellular Uptake

#### 2.7.1. Preparation of Fluorescent Probe

Loaded NPs were further labeled with a coumarin-6 fluorescent probe (C6; the dosage was 1 mg). The generated C6 NPs were used to determine the cellular uptake of the particles by fluorescence microscopy imaging. 4T1 and A549 cells were seeded at 1 × 10^5^/well in six-well plates and cultured for more than 24 h until 80% cell coverage was achieved. Solutions with various modified nano-drug concentrations were added separately and incubated for 4 h.

#### 2.7.2. Qualitative Study of Cellular Uptake

The cellular uptake of the drugs (serum-free medium with diluted free-C6 solution and C6 NP, C6 PD NP, and C6 PD-RGD NP solutions with a C6 concentration of 1 μg/mL) was qualitatively determined using inverted fluorescence microscopy (DMi8, LEICA, Wetzlar, Germany). The cells, fixed with 4% paraformaldehyde and then stained with DAPI, were observed using inverted fluorescence microscopy.

#### 2.7.3. Quantitative Study of Cellular Uptake

The cellular uptake of the drugs was determined quantitatively using flow cytometry (Attune, Thermo Fisher Scientific, Carlsbad, CA, USA) for serum-free medium dilutions of C6 NP, C6 PD NP, and C6 PD-RGD NP solutions with a C6 concentration of 1 μg/mL. After incubation, the cells were washed twice with PBS, harvested using trypsin treatment, fixed with 4% paraformaldehyde, and resuspended in PBS for flow cytometry. Flow cytometry data were processed using FlowJo v10.8.1 software.

### 2.8. Biocompatibility Assay

4T1 and A549 cells were seeded in 96-well plates at 5 × 10^3^/mL in 100 μL of DMEM containing 10% FBS. After 24 h of incubation, 100 μL of the blank nanocarrier was added to the 96-well plates, and the plates were incubated for another 24 and 48 h. At the end of the incubation, the drug-containing medium was removed from the wells, and then 100 μL of fresh DMEM and 10 μL of CCK8 were mixed and added to each well, followed by incubation for 4 h. The OD of each well was measured at 450 nm using a microplate reader (800TS, BioTek, Winooski, VT, USA).

### 2.9. In Vitro Cytotoxicity Assay

4T1, A549, HeLa, and MCF-7 cells were seeded in 96-well plates at 5 × 10^3^/mL in 100 μL of DMEM containing 10% FBS. After 24 h of incubation, 100 μL of samples at different concentrations was added to the 96-well plates and incubated for another 24 h (samples: commercially available paclitaxel injection (positive control), PTX NPs, PTX PD NPs, and PTX PD-RGD NPs). After the incubation, the drug-containing medium was removed from the wells, and 100 μL of fresh DMEM and 10 μL of CCK8 were mixed and added to each well, followed by incubation for 0.5~2 h. The OD of each well was measured at 450 nm using a microplate reader (800TS, BioTek, Winooski, VT, USA).

### 2.10. Ethics Approval

Female BALB/c mice (19 ± 1 g, 3–5 weeks of age) were supplied by the Guangdong Medical Laboratory Animal Center (Guangzhou City, China). The animals were acclimatized at 25 ± 2 °C and a relative humidity of 70 ± 5% under natural light/dark conditions for one week before dosing. All animal experiments were approved by the Institutional Animal Care and Use Committee of Guangdong Pharmaceutical University (application number: GDPULUU2021202).

### 2.11. Tumor Model Establishment

A tumor-bearing mouse model was prepared by injecting 5 × 10^5^ 4T1 cells subcutaneously into the right back of female BALB/c mice. The animals were checked daily for tumor development at the site of injection.

### 2.12. In Vivo Antitumor Activity Evaluation of PLGA NPs in Tumor-Bearing Mice

After the tumor volume increased to approximately 200 mm^3^, 6 mice excluded from modeling were assigned to the normal group, and 24 tumor-bearing mice were randomly divided into four groups of 6 each and treated as follows:

- Normal group: 0.1 mL of physiological saline per 10 g;
- Negative control (model) group: 0.1 mL of physiological saline per 10 g;
- Positive control group: commercially available paclitaxel injection (10 mg/kg);
- PTX PD NP group: without active-target-modified delivery NPs (10 mg/kg);
- PTX PD-RGD NP group: with active-target-modified drug delivery NPs (10 mg/kg).

All drugs were administered by tail vein injection every other day for four doses. Mice were weighed daily, and tumor volumes were measured. At the end of dosing, after weighing each group of mice and measuring the tumor volumes, blood was collected from the orbits for routine blood and blood biochemistry tests, and the mice were sacrificed.

The weight suppression rate, tumor volume suppression rate, and liver, spleen, kidney, and thymus indices were calculated, and tumor volume changes were observed in mice. Tumors were collected in 4% paraformaldehyde and used to prepare hematoxylin–eosin staining (HE), Ki67, and TUNEL pathological sections for testing.

## 3. Results and Discussion

### 3.1. Characterization of PTX PD-RGD NPs

**Figure 2:**
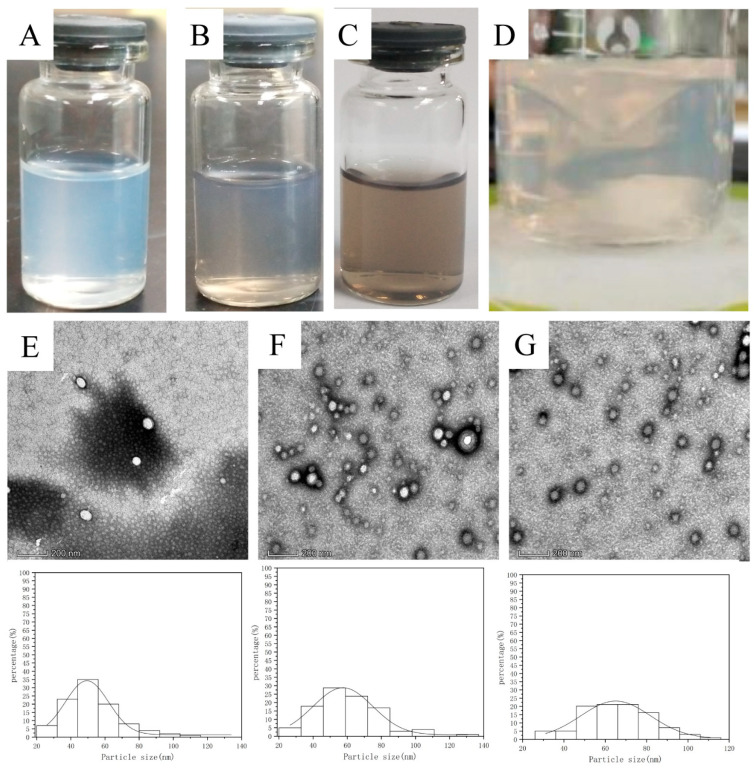
Characterization of NPs ((**A**): PTX NPs; (**B**): PTX PD NPs; (**C**): PTX PD-RGD NPs; (**D**): indole intermediate). Transmission electron micrograph of NPs and Nano Measurer v1.2 software analysis of particle size distribution ((**E**): PTX NPs; (**F**): PTX PD NPs; (**G**): PTX PD-RGD NPs).

The appearance of the three different NP solutions (PTX NPs, PTX PD NPs, and PTX PD-RGD NPs) obtained according to different modification methods was as follows: The PTX NP solution was light blue, opalescent, clarified, and transparent. The PTX PD NP and PTX PD-RGD NP solutions were light brown, opalescent, clarified, and transparent. Dopamine underwent a self-polymerization reaction and was encapsulated in the NP master nuclei in a weakly alkaline environment, changing from a light-pink indole intermediate (Figure 2D) to the light brown color of PD; the final PD-modified NPs exhibited brown opalescence.

The physicochemical characteristics of the PTX NPs, PTX PD NPs, and PTX PD-RGD NPs are shown in Table 1, showing differences in particle size (nm), PDI, zeta potential (mV), and EE (%).

The particle size analysis showed that the size of all investigated NPs was in the range of 134.5 ± 0.9 to 137.6 ± 2.9 nm. The homogeneity of the size distribution, indicated by PDI values of <0.300, was acceptable. The slight increase in particle size distribution and D90 values of PTX PD NPs compared to PTX NPs was due to the minute thickness of the nano-coat formed by PD at low PD concentrations of 0.5 mg/mL under weakly alkaline conditions during the 3 h coating of the substrate. In agreement with the results already reported [44,45], the particle size increased by approximately 2 nm as a result of PD modification. The RGD peptide modification resulted in little change in particle size and a broadened particle size distribution, probably due to the low-molecular-weight RGD grafted onto the PD-coated surface.

The catechol structure contained in PD causes the NPs to be negatively charged; strong electrostatic repulsion causes the PD-wrapped NPs to exhibit strong stability. The NPs had a moderate negative charge that protects them from being adsorbed in vivo by substances such as plasma proteins and prevents their deposition on vessel walls; however, the negative charge density should not be infinitely large so as not to repel platelets to the vessel wall to aggregate and cause thrombosis [47]. The three NPs had negative surface charges, with absolute values ranging from 5 to 25 mV, and the system was relatively stable. The absolute value of the ζ-potential decreased from the pre-modification value, indicating that the system tended to agglomerate more easily. The NPs could be prepared as a lyophilized powder for easy transportation and storage.

The encapsulation rate of all three NPs was above 80%. The PD coating increased the encapsulation rate of the NPs, presumably because it encapsulated the paclitaxel embedded in the PLGA carrier or adsorbed on the PLGA surface, thus increasing the drug-loading capacity. The NP content after RGD modification was slightly reduced when the same mass of NPs was measured.

The three NPs were spherical. The PD-modified NPs had a more regular morphology and were covered with a thin-film-like material. The recorded TEM images were analyzed using the Nano Measurer software, and the sizes of the NPs in the images were counted. The size distributions of the three NPs were in the range of 20–120 nm. The particle size observed using TEM was smaller than that measured by the laser particle size analyzer, likely owing to bulk effects in the liquid form.

### 3.2. Solid-State Characterization

#### 3.2.1. DSC Characterization

**Figure 3:**
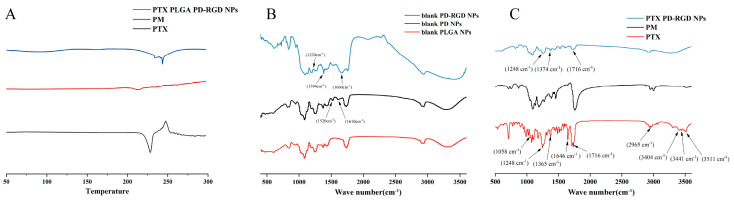
(**A**): DSC spectrum; (**B**): infrared spectrum of blank NPs; (**C**): infrared spectrum (PTX: paclitaxel API; PM: paclitaxel and PLGA physical mixture; NPs: RGD-modified paclitaxel-loaded NPs).

DSC was used to detect the form of the drug present in the nanomedicine. The DSC thermal profile in Figure 3A shows that paclitaxel exhibited sharp heat absorption and exothermic peaks attributed to the melting of paclitaxel, indicating its crystalline nature. The DSC curve for the physical mixture of the drug and carrier material showed a heat absorption peak, probably due to the melting of the polymer in the mixture and the low paclitaxel content; therefore, the heat absorption exothermic peak was not obvious. In contrast, drug-carrying NPs showed only a broad heat absorption peak, suggesting that paclitaxel was in an amorphous state, which is conducive to improving the stability of the drug.

#### 3.2.2. FTIR Characterization

FTIR spectroscopy was used to verify the PD coating and the modification of RGD. Figure 3B shows that PD NPs had increased peak bands at 1610 cm^−1^ and 1520 cm^−1^ compared to PLGA NPs, consistent with the C=C peak of PD. The increased peak band at 1660 cm^−1^ after RGD modification compared to the PD NPs is consistent with the Schiff base reaction’s stretching vibrational peak of –C=N. The 1200 cm^−1^ peak band was consistent with the –C–C–N peak of RGD, and the 1384 cm^−1^ peak band was consistent with the bending vibration peak in the –C–H plane of RGD, suggesting that RGD couples to the PD surface via a Michael addition reaction with the PD coating at 1200 cm^−1^. These results illustrate the successful coupling of RGD to the PD-coated surfaces.

Figure 3C shows that the main characteristic absorption peaks of paclitaxel were 3511, 3441, and 3404 (–OH vibration peak), 2965 (=C–H carbon–hydrogen bond), 1716 (C=O carbonyl ketone stretching vibration), 1248 and 1646 (CONH_2_ amide group stretching vibration), 1365 (CH_3_ methyl bending vibration), and 1058 cm^−1^ (C–O stretching vibration); the same characteristic absorption peaks existed in the physical mixture, indicating that no obvious chemical interactions occurred between paclitaxel and the carrier. The same characteristic absorption peaks were observed in the nanoparticle spectrum, i.e., 1716, 1248, and 1374 cm^−1^ (CH_3_ methyl bending vibration), indicating that paclitaxel did not chemically interact with the carrier material.

#### 3.2.3. XPS Characterization

**Figure 4:**
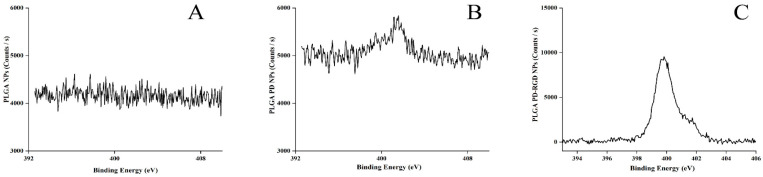
Elemental analysis map of three NPs ((**A**): PLGA NPs; (**B**): PLGA PD NPs; (**C**): PLGA PD-RGD NPs).

Surface elemental analysis was performed using XPS (Figure 4). The PLGA NPs did not contain nitrogen, so there was no corresponding peak in the high-resolution spectrum of NPs, while the addition of PD to the NP surface induced a peak corresponding to nitrogen, directly proving the successful coating of PD on the NP surface.

RGD contains nitrogen; therefore, after regrafting, the concentration of the RGD modification was higher, so the peak height increased significantly. The nitrogen content increased, successfully demonstrating the modification of RGD on the surfaces of the NPs.

### 3.3. In Vitro Drug Release

**Figure 5:**
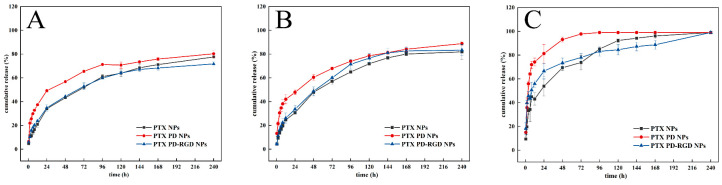
In vitro release curves of NPs under different pH conditions ((**A**): pH 7.4; (**B**): pH 6.8; (**C**): pH 5.2).

The three release profiles (Figure 5) show that the cumulative release rate of all groups of NPs under all pH conditions exceeded 80%. The cumulative release rate of all groups of NPs at pH 5.2 was significantly greater than that of the NP groups under other pH conditions, reaching over 90% with complete release. At pH 7.4, the cumulative release of NPs was significantly lower than at pH 6.8 and 5.2 for all groups, and the release was slow for all groups. Compared with the PLGA NPs, the PD-coated NPs had smoother release profiles.

Notably, the NP release was greater under slightly acidic conditions, such as those typically found at tumor sites [4], suggesting a certain pH effect that would enable the NPs to better target the tumor site. PD NPs at pH 5.2 showed faster release at first. PD structures contain active double bonds that can chemically react with several groups, and a large number of sulfhydryl compounds can degrade the PD shell; thus, PD-encapsulated NPs can be degraded by glutathione when they enter the cells [48]. The PD coating’s pH-sensitive nature [49,50] allows for the release of paclitaxel adsorbed on or embedded in PLGA, such that the release of PTX PD NPs exhibits a burst release, followed by a delayed release. The binding of RGD to the PD-coated surface was demonstrated indirectly. The three release profile plots also illustrate the pH-responsive release properties of the PD coating on the NPs, with a slow release of the drug in a neutral environment and more rapid release under acidic conditions. The equations were further fitted to the release behavior of each group of NPs under each pH condition using OriginPro 2018 v9.5.1 software. The PD-modified NPs were found to exhibit Ritger–Peppas release at pH 6.8 and 5.2 with a diffusion coefficient of *n* < 0.45, exhibiting Fickian diffusion, probably because of the easy depolymerization of PD and accelerated hydrolysis of PLGA [51] in the acidic microenvironment of the tumor site, which facilitates the release of the drug from the NP carriers.

### 3.4. Cellular Uptake

Researchers have found that NPs with a size of approximately 100 nm enter the cell mainly through the clathrin-mediated endocytic (CME) pathway [52]. Paclitaxel is commonly used clinically to treat breast and non-small-cell lung cancers; therefore, uptake research uses these two types of tumor cells. The specific uptake of NPs by tumor cells is a key step in drug efficacy. NPs were prepared using coumarin-6 instead of paclitaxel, and the cellular uptake of the NPs was observed.

**Figure 6:**
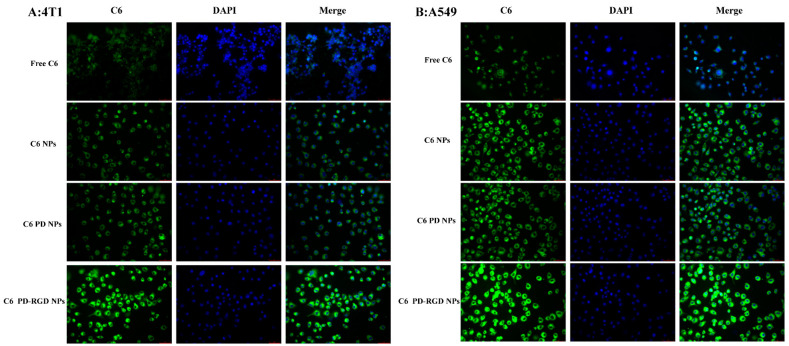
Uptake of coumarin NPs by 4T1 (**A**) and A549 (**B**) cells under an inverted fluorescence microscope (magnification: 40 × 10) (C6 is shown in the coumarin fluorescence diagram, showing coumarin green fluorescence; DAPI is shown in the nuclear-staining diagram, showing nuclear blue fluorescence; the merge diagram shows the superposition of the C6 and DAPI fluorescence diagrams, showing the mixed color of blue nuclei and green coumarin NPs).

The experimental results (Figure 6) show that green C6 fluorescence was distributed in the blue nuclei of cells. Green fluorescence was barely observed around the nuclei of cells in the free-C6 group, whereas noticeable fluorescence was observed around the nuclei of cells in the C6 NP, C6 PD NP, and C6 PD-RGD NP groups.

The fluorescence intensity of the merged fluorescence images was further analyzed using ImageJ Fiji v2.10.0 software. The fluorescence intensity of the C6 PD-RGD group was significantly higher than that of the other NP groups (*p* < 0.01), suggesting that the RGD modification enhanced the uptake of NPs by tumor cells.

**Figure 7:**
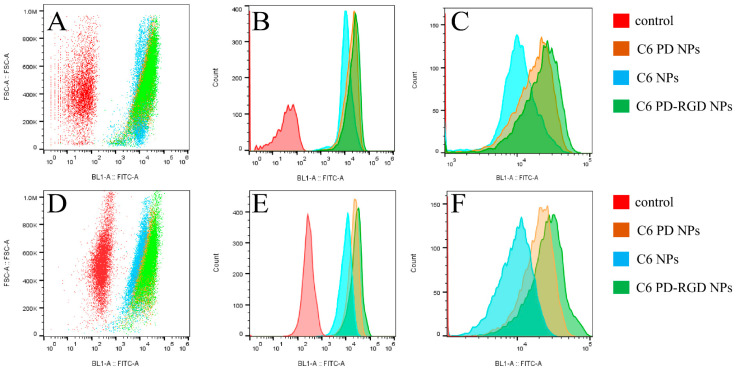
Uptake of coumarin NPs by 4T1 cells was measured using flow cytometry ((**A**): distribution of fluorescent cells; (**B**): displacement of fluorescent cells; (**C**): local magnification of fluorescent cell displacement). The uptake of coumarin NPs by A549 cells was measured using flow cytometry ((**D**): distribution of fluorescent cells; (**E**): displacement of fluorescent cells; (**F**): local magnification of fluorescent cell displacement).

The uptake of the three NPs by these two cell types was quantitatively analyzed using flow cytometry (Figure 7). Compared to the blank control group, all three NPs showed stronger, significantly displaced fluorescence intensity; the fluorescence intensity reached more than 1 × 10^4^. The local magnification of the three NP displacement plots by horizontal-axis FITC-A showed that in both cell types, the fluorescence intensity of RGD-modified NPs was significantly higher than that of PD and PLGA NPs.

**Figure 8:**
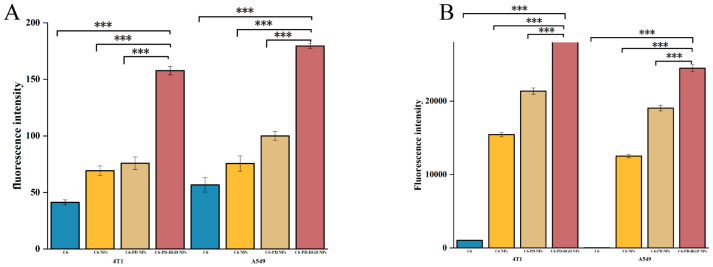
Analysis of mean fluorescence intensity ((**A**): results of ImageJ Fiji v2.10.0 software analysis of the captured images; (**B**): quantitative results of FlowJo v10.8.1 software analysis of the intake compared with RGD NPs; *** *p* < 0.001; *n* = 3).

The analysis of the mean fluorescence intensity (Figure 8) showed that the uptake of RGD-modified coumarin NPs in both tumor cell lines reached approximately 3 × 10^4^ fluorescence intensity, more than twice that of PLGA NPs and PD NPs, indicating that RGD active-targeting modification promoted the uptake of the nano-drug by tumor cells. The quantitative uptake results also showed that the uptake of PD-modified NPs by both tumor cell types was higher than that of PLGA NPs, possibly due to the higher hydrophilicity of the PD coating on the NPs [53].

### 3.5. In Vitro Cytotoxicity Assay

**Figure 9:**
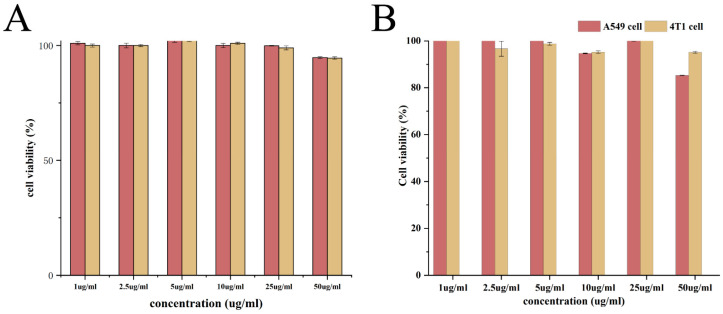
Cytotoxicity of blank nanocarriers ((**A**): nanocarrier incubated for 24 h; (**B**): nanocarrier incubated for 48 h).

Human-derived murine tumor cells were co-incubated with PLGA PD-RGD NP nanocarriers in a concentration gradient for 24 h and 48 h. The cell survival rate was above 80% (Figure 9), indicating that the studied nanocarrier materials had good biosafety.

**Figure 10:**
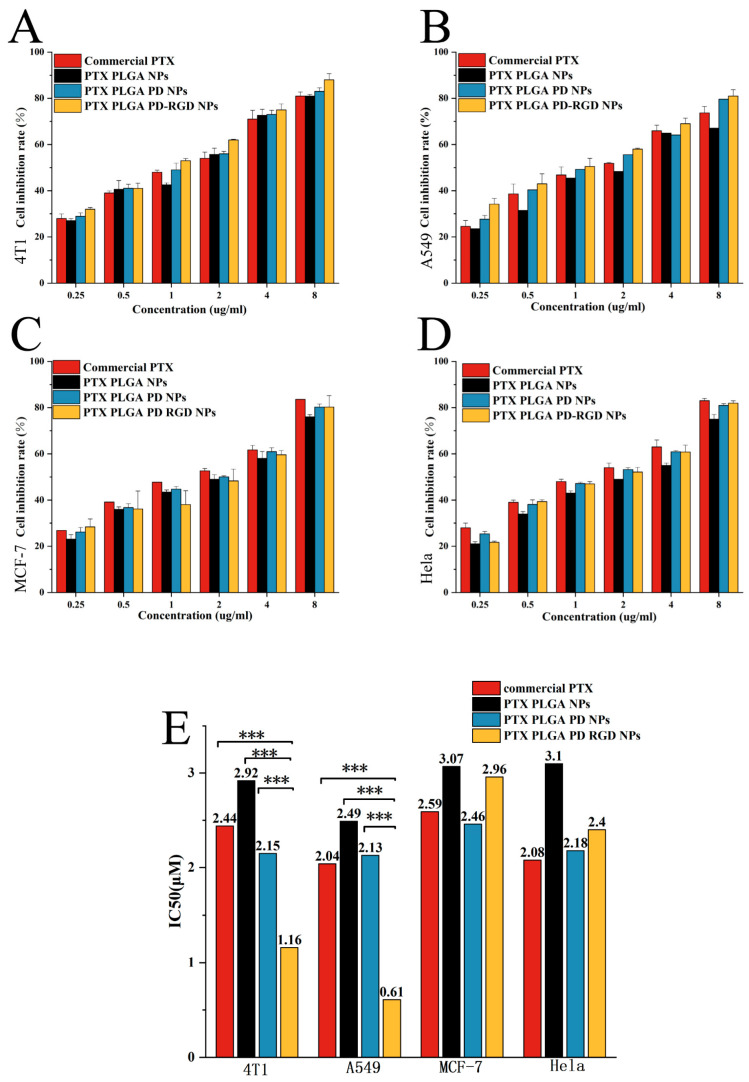
(**A**–**D**): Cytotoxicity of paclitaxel formulation on various tumor cells; (**E**): IC_50_ values of paclitaxel formulation on various tumor cells (compared with the RGD-modified NP group, *** *p* < 0.001).

The cytotoxic effects of paclitaxel NPs were quantified using commercially available paclitaxel as a positive control and compared with those of PTX NPs, PTX PD NPs, and PTX PD-RGD NPs. In all four tumor cell lines (Figure 10), RGD-modified NPs showed better tumor cell growth inhibition than commercially available formulations, with IC_50_ values below 3 μM. In all groups of cells, the IC_50_ values of PD-coated NP cells were lower and more cytotoxic compared to PLGA NPs, probably due to the accelerated drug release after PD modification and the higher cellular uptake and entry of the nano-drug into tumor cells.

The higher IC_50_ values in the PTX NP group compared to commercially available preparations were attributed to the time required for paclitaxel release from the PLGA NP shell and lower drug release during the 24 h incubation time, while the commercially available paclitaxel preparations directly interacted with the cells. In 4T1 and A549 cells, with high integrin αvβ3 receptor expression, RGD-modified NPs showed higher cytostatic rates and significantly lower IC_50_ values than the commercially available preparation.

In A549 cells, the IC_50_ of the commercially available formulation of paclitaxel was 2.04 μM. In comparison, the IC_50_ of RGD-modified NPs was significantly different at 0.61 μM (*p* < 0.001) and three-fold smaller than that of the commercially available formulation. This may be because RGD-modified NPs with active targeting entered a greater number of tumor cells, enhancing the antitumor efficacy. For the 4T1 cells, the IC_50_ value of the commercially available formulation was 2.44 μM, and the RGD-modified NPs had an IC_50_ value of 1.16 μM, approximately two-fold less than the IC_50_ value of the commercially available formulation (*p* < 0.001). As for MCF-7 and HeLa cells, with low expression of the integrin αvβ3 receptor, the cytostatic rate of commercially available preparations and the IC_50_ value of RGD-modified NPs were approximately 2 μM. Daniel et al. [54] have recently shown that soft fluorescent organic nanoparticles do not significantly inhibit cell survival because integrin αvβ3 is overexpressed in glioblastoma cells and responsible for the preferential uptake of nanoparticles in U-87 MG cells. These findings are consistent with our experimental results.

### 3.6. In Vivo Antitumor Study

**Figure 11:**
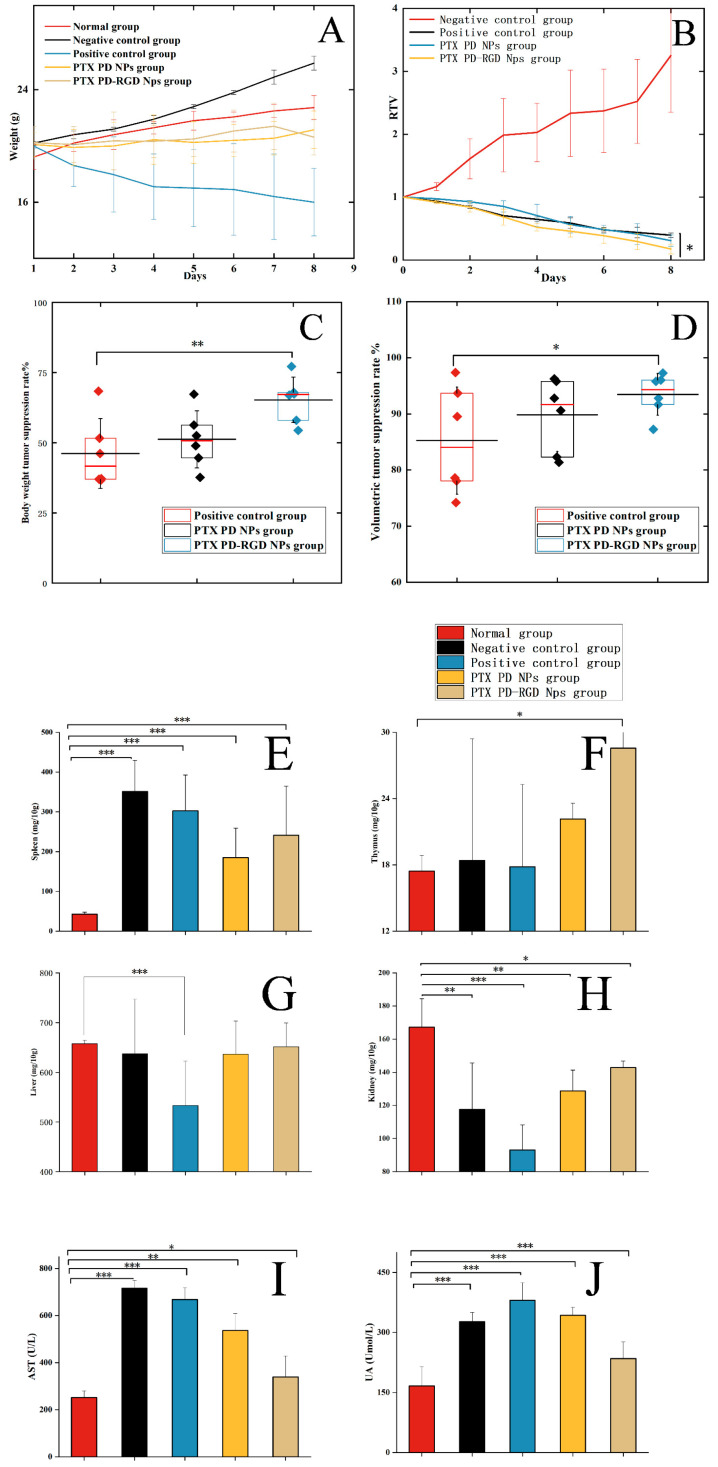
(**A**): Changes in body weight of mice in each group after administration; (**B**): changes in tumor volume of mice in each group; tumor suppression rate of drug administration group; (**C**): weight suppression rate of drug administration group; (**D**): tumor volume suppression rate of drug administration group. Organ index and thymus index of mice in each group after 8 days of administration ((**E**): spleen index; (**F**): thymus index; (**G**): liver index; (**H**): kidney index; (**I**): AST; (**J**): UA; compared with the normal group, * *p* < 0.05, ** *p* < 0.01, *** *p* < 0.001).

During the modeling experiments, the condition of the tumor-bearing mice deteriorated; the whole-body hair color lacked luster and was accompanied by hair loss. These effects were most obvious in the negative and positive control groups, and the overall condition of the NP group was relatively good. Comparing the weight changes among the groups (Figure 11A), the weight of the negative control group increased significantly, with an increase of 28.5%. In contrast, the body weight of the positive control group decreased. The rate of weight loss was 25%, which may be an adverse reaction to injecting a commercially available paclitaxel drug. The results reflect the improved in vivo biosafety of the NP administration group, which could alleviate the adverse drug reactions of paclitaxel.

The routine blood test results for the mice in each group are shown in Table 2. Compared to the normal group, the number of leukocytes in the blood of all other groups was significantly increased (*p* < 0.001). It was significantly higher than the normal range, indicating that the tumor-bearing mice showed an immune response to tumors in vivo. Lymphocytes are responsible for clearing tumor cells; when the number of lymphocytes decreases, the chance of immune escape increases, leading to tumor development and distal metastasis. The number of lymphocytes increased in the NP administration group. In contrast, the number of lymphocytes increased to a lesser extent in the positive administration group. The increase in lymphocytes was more evident in the PD-modified NP group.

The neutrophil–lymphocyte ratio (NLR) reflects the prognosis of cancer. An increase in the ratio predicts a worse prognosis and shorter survival in tumor patients. The positive control group had a higher ratio than the other groups, indicating a poorer prognosis after treatment.

Advanced breast cancer is usually accompanied by blood hypercoagulation and hemostasis disorders; consistently, platelet counts were elevated in the negative control group of tumor-bearing mice. After treatment, all groups of mice showed varying degrees of decrease in platelet count toward the normal range, compared with the normal group and the NP administration group. The positive administration group showed a lower platelet count, probably due to the adverse effects of paclitaxel.

The spleen and thymus are two important immune organs. The spleen and thymus indices were calculated for each group of mice (Figure 11E–J). The spleen index was significantly higher in tumor-bearing mice than in the normal group (*p* < 0.01), indicating that the mice activated their own immune response to the tumor. The thymus index increased to varying degrees in the NP-treated group, consistent with the increased number of lymphocytes.

The liver index showed some degree of liver damage in the positive administration group compared with that in the normal group. The renal index of each dosing group was significantly lower than that of the normal group, indicating that all dosing groups showed different degrees of renal damage, with the positive control group showing the most significant damage (*p* < 0.001). This indicates that the toxicity of paclitaxel can be reduced to a certain extent when paclitaxel is delivered via NPs.

Liver- and kidney-related blood biochemical indices were determined to examine the extent of liver and kidney damage. The blood biochemistry results are shown in Figure 11I,J. Aspartate aminotransferase (AST) indicates liver function, with a normal range of 36.31~250.48 U/L. Compared to the normal group, all other groups showed different degrees of AST elevation, suggesting that the tumor-bearing mice had different degrees of liver injury. The negative control and positive control groups showed significant AST elevation, whereas the NP administration group showed a lower degree of liver injury.

Uric acid (UA) is used to evaluate kidney function, with a normal range of 44.42~224.77 Umol/L. The UA index of the tumor-bearing mice was significantly higher than that of the normal group, suggesting that kidney and urinary tract abnormalities may have existed in the tumor-bearing mice. In contrast, damage was alleviated in the NP-administered group [55,56].

Taken together, body weight changes, blood tests, and various organ and biochemical indices in the mice indicated that the PD-RGD NP administration group had better biosafety than the commercially available formulation.

Each group’s relative tumor volume change curves after eight days of administration are shown in Figure 11B. After 8 days of administration, the mice were sacrificed, and the tumor tissues were removed. The relative tumor volume was compared with the tumor volume of mice before administration. The higher dose of short-term administration in the administered group improved tumor growth inhibition compared with the negative control group; the tumor-bearing mice had good sensitivity to paclitaxel and did not develop drug resistance in the short term. Compared to the commercially available paclitaxel and PTX PD NP administration groups, the tumor suppression effect was more pronounced in the RGD-modified NP administration group (*p* < 0.05). The increased tumor suppression rate in the PD-RGD NP group at the later administration stage may have been due to the accumulation of NPs in the tumor tissue.

The box line plots of the tumor volume suppression and weight suppression rates (Figure 11C,D) show rates of 65% in the PTX PD-RGD NP administration group and 46% in the positive administration group, with a significant difference (*p* < 0.01) and a more uniform and concentrated tumor weight distribution. The tumor volume suppression rate reached 90% in the PTX PD-RGD NP administration group and 80% in the positive administration group, which was statistically different (*p* < 0.05). The tumor volume distribution was more uniform and concentrated in the NP administration group, consistent with the isolated tumor outcome map.

The median tumor suppression rate in the NP group was higher than in the positive control group, with the tumor volume suppression rate being higher. The PD-RGD NP-treated group exhibited superior tumor suppression, attributed to the tumor-targeting effect of RGD. The effect in the PD NP administration group was slightly better than that in the commercially available formulation group, probably because of the greater uptake of PD NPs by tumor cells.

HE staining presents an image of cells with blue nuclei and red cytoplasm. Ki67 is actively expressed during cell proliferation but is silent during proliferation arrest; therefore, it can be used as a signal of the cell proliferation status to determine the malignancy of tumors. The higher the Ki67 expression rate, the stronger the proliferative activity of tumor cells, the higher the malignancy, and the higher the risk of tumor recurrence and metastasis. The TUNEL signal indicates apoptosis by detecting the exposed 3’-OH end of the DNA. Both apoptotic and necrotic cells with damaged DNA and exposed 3’-OH ends generate TUNEL signals.

**Figure 12:**
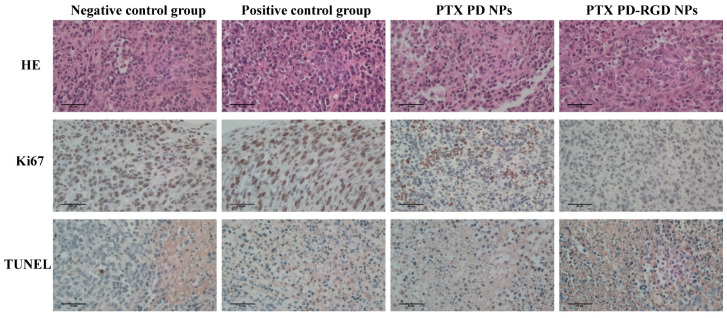
HE, Ki67, and TUNEL analysis of mice in each group.

The results of Ki67 staining (Figure 12) showed the highest expression in the negative control group, lower expression in the other groups, and weakly positive results (brown, yellow, and tan), indicating that paclitaxel could inhibit the proliferation of tumor cells, consistent with the pharmacological mechanism of paclitaxel. The modification of NPs with RGD could enhance the inhibition of tumor cell proliferation. TUNEL assay results showed that apoptosis was induced in the drug administration groups, and the positive results were stronger in the RGD-modified NP group compared with that in the paclitaxel group.

The ImageJ IHC profiler plug-in was used to score the tumor-stained section images (Table 3) automatically. For the Ki67 sections, the negative control group was positive, the positive control and PD NP groups were weakly positive, and the PD-RGD NP group was positive. In the TUNEL section group, the negative control group was negative, the PD NP group was weakly positive, and the PD-RGD NP and positive control groups were positive, all consistent with the results observed in the images.

The tumor suppression rate and tumor-factor-related expression data indicated that the RGD-modified NPs showed a superior tumor suppression effect compared to that of the commercially available formulation; this could be attributed to the successful PD-mediated secondary modification of the NP surface. In vivo anti-breast-cancer studies preliminarily indicated that the prepared PTX PD-RGD NPs have potentially fewer toxic effects.

## 4. Conclusions

In this study, we used a PD-mediated surface modification strategy to successfully construct paclitaxel-loaded PLGA NPs modified with PDA-bridged RGD peptides. Unlike traditional NP surface modification methods, our method conferred new surface properties to the NPs. The relevant properties of the produced nanomedicines were systematically evaluated, and the experimental results showed that they initially achieved the delivery of chemotherapeutic drugs with increased efficacy and reduced toxicity, providing a reference for PD-modified nanomedicine that can be used as a potential carrier platform for targeted drug delivery. However, the present study had limitations. The relationship between the process conditions of PD coating and the amount of RGD coupling in the preparation process needs to be studied in depth to achieve the optimal drug delivery effect. The photothermal conversion performance of the PD coating can be further combined with photothermal therapy to maximize the benefits that PD nanocoatings offer. Simultaneously, the focus should be on the in vivo metabolic pathway of PD to fill the gaps in our understanding of the in vivo metabolic fate of PD as a nano-drug carrier.

## Figures and Tables

**Table 1:** Characterization of the three nanoformulations (*n* = 3).

Preparation	Size (nm)	D90 (nm)	PDI	ζ (mV)	EE (%)
PTX NPs	134.5 ± 0.9	227.6 ± 11.8	0.108 ± 0.018	−15.63 ± 2.34	85.06% ± 0.18
PTX PD NPs	136.8 ± 0.4	230.6 ± 7.4	0.136 ± 0.030	−12.11 ± 1.82	87.31% ± 0.43
PTX PD-RGD NPs	137.6 ± 2.9	232.9 ± 11.7	0.104 ± 0.010	−8.20 ± 1.24	81.72% ± 0.49

**Table 2:** Routine blood test results of each group (compared with the normal group, *** *p* < 0.001).

Category	Units	Normal Group	Negative Control Group	Positive Control Group	PTX PD-RGD NP Group	PTX PD NP Group	Reference Range
Leukocyte	10^9^/L	8.3 ± 1.4	217.6 ± 71.9 ***	225.7 ± 46.4 ***	220.9 ± 88.4 ***	254.1 ± 69.9 ***	0.8–8
Lymphocyte	10^9^/L	6.40 ± 2.10	84.6 ± 32.52 ***	35.03 ± 29.89 ***	74.63 ± 35.07 ***	113.8 ± 26.00 ***	0.7–6.0
Monocyte	10^9^/L	0.2 ± 0.1	9.4 ± 4.6 ***	9.7 ± 4.9 ***	9.7 ± 3.7 ***	12.6 ± 4.6 ***	0.0–0.3
Neutrophil	10^9^/L	1.7 ± 1.1	144.1 ± 104.9 ***	180.9 ± 13.1 ***	136.5 ± 95.3 ***	103.5 ± 68.8 ***	0.1–1.8
Lymphocyte percentage	%	74.9 ± 14.8	50.8 ± 39.8	14.2 ± 9.3 ***	52.7 ± 5.8	57.6 ± 19.8	55.8–90.6
Neutrophil/lymphocyte	--	0.27 ± 0.21	1.70 ± 2.10	5.17 ± 4.10 ***	3.15 ± 2.71	0.83 ± 0.88	--
Erythrocyte	10^12^/L	8.50 ± 4.08	7.53 ± 1.65	9.42 ± 1.50	8.32 ± 0.36	6.90 ± 0.73	6.36–9.42
Thrombocyte	10^9^/L	1025 ± 79	1845 ± 768 ***	619 ± 81	778 ± 114	894 ± 350	450–1500

**Table 3:** Scoring results for stained tumor sections.

Score	Negative Control Group	Positive Control Group	PTX PD NPs	PTX PD-RGD NPs
Ki67	Positive	Low Positive	Low Positive	Negative
TUNEL	Negative	Low Positive	Low Positive	Positive

## Data Availability

Data are contained within the article.
